# Monitoring Allergen Immunotherapy Effects by Microarray

**DOI:** 10.1007/s40521-016-0084-2

**Published:** 2016-04-20

**Authors:** Christian Lupinek, Eva Wollmann, Rudolf Valenta

**Affiliations:** Division of Immunopathology, Department of Pathophysiology and Allergy Research, Center for Pathophysiology, Infectiology and Immunology, Medical University of Vienna, Waehringer Guertel 18-20, 3Q, 1090 Vienna, Austria

**Keywords:** Allergy, Recombinant allergen, Allergen-specific immunotherapy, Allergen-microarray, Monitoring, Biomarker, Blocking antibodies

## Abstract

Allergen-specific immunotherapy (AIT) is the only treatment of IgE-mediated allergies so far that has a sustained effect on clinical symptoms and can modify the course of the disease. It is an allergen-specific treatment and therefore requires the correct identification of the disease-causing allergens. Furthermore, AIT is a time-consuming treatment for which the efficacy is dependent on several factors. Therefore, diagnostic tests and biomarkers are needed that facilitate (1) selection of the correct allergens according to the patient’s individual sensitization profile and (2) to monitor the effects of AIT. This can provide support for the decision to continue, modify, or discontinue vaccination. One significant mechanism of action of AIT is the induction of allergen-specific antibodies that compete with IgE for the binding to allergen molecules, hence referred to as blocking antibodies. It was shown in several studies that the induction of blocking antibodies by AIT, and their specificity can be measured by allergen microarrays. Inhibition of allergen-specific IgE binding by blocking antibodies can also be determined by microarrays and is associated with changes in clinical parameters or other in vivo and in vitro assays demonstrating efficacy of AIT. Furthermore, allergen microarrays allow determination of IgE sensitizations towards a comprehensive set of allergen molecules and therefore are well suited for identifying the disease-causing allergens for correct prescription of AIT. Thus, diagnostic tests based on microarrayed allergens can be useful in determining the correct prescription of AIT and can be used to monitor efficacy of AIT.

## Introduction

During the last decades, the prevalence of IgE-associated allergies increased worldwide, affecting currently millions of patients, some of whom suffering from severe or even life-threatening conditions [1–7]. Analysis of approximately 6500 sera from population-based European birth cohorts in the course of the MeDALL project [8] indicates an even higher percentage of sensitized children which most probably will lead to higher prevalence of allergic disease in the decades to come. Symptomatic medications like antihistamines, mast cell stabilizing agents, leukotriene receptor antagonists or, in more severe cases, corticosteroids or anti-IgE antibodies only have short-term effects and need to be administered regularly which causes considerable costs and burden to the patients due to adverse effects of the drugs. Allergen immunotherapy (AIT) is a cost-effective therapy and, so far, the only treatment that can yield sustained symptomatic improvement [9]. However, there are several factors that may hamper clinical efficacy of AIT, some of which are directly associated with the use of allergen extracts for vaccination. Due to the great variability of natural allergen sources regarding allergen composition and concentration, allergen extracts used both for diagnosis and therapy show considerable variation when products from different producers or different batches are compared [10–14]. In addition, specimens from particular allergen sources may contain clinically relevant allergens but their amounts are insufficient, e.g., Der p 23 from house dust mite [15], or are in general difficult to extract, like material from fungi [16, 17]. Therefore, vaccines for AIT that are based on natural allergen extracts often do not cover the individual sensitization profile of the patient in terms of allergen composition and thus, treatment may fail in such cases. However, even if the vaccine does contain all clinically relevant allergen molecules it is not possible to predict for the individual patient if AIT is likely to induce a beneficial immune response because certain allergens may exhibit low immunogenicity and/or there may be non-responders among patients.

Allergen immunotherapy is a treatment which requires considerable patient time and health care resources. Albeit in general it is very safe, there is the risk of severe systemic side effects [18]. It has been suggested that accuracy of prescription of AIT can be improved by component resolved diagnosis [19–21] which was confirmed by an increasing number of studies [22–24]. Therefore, diagnostic algorithms based on molecular diagnosis have been developed for several respiratory and venom allergies [25–27].

## Evidence for the role of blocking antibodies for clinical efficacy of AIT

**Fig. 1:**
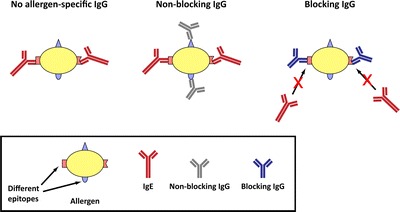
Blocking versus non-blocking antibodies. Non-blocking allergen-specific IgG antibodies (*gray*) recognize different epitopes than IgE antibodies (*red*) specific for the same allergen molecule and therefore both IgE and IgG can bind simultaneously to the same allergen. In contrast, blocking IgG antibodies (*blue*) bind to the same region as allergen-specific IgE and therefore, competition between IgE and IgG for binding to the same molecule occurs.

In 1911, the first allergen-specific immunotherapy (AIT) study was published by Leonard Noon [28]. His work was inspired by the demonstration that antisera can be raised against pollen allergens in animals which could neutralize their allergenic activity when applied to allergic patients, a finding which already emphasized the importance of protective antibodies for preventing allergic symptoms [29]. Carl Prausnitz and Heinz Küstner demonstrated that reactivity to allergens can be specifically transferred by intradermal injection of sera obtained from allergic subjects into the skin of healthy individuals or of subjects allergic to other allergen sources [30]. This experiment identified a serum factor specific for allergens which later was identified as immunoglobulin E as being responsible for allergic reactions [31] and paved the ground for further investigations of mechanisms underlying AIT. Using the approach of passive serum transfer of Prausnitz and Küstner, Cooke showed that AIT induced a blocking antibody response in treated patients which could suppress allergic reactions [32]. In these experiments, sera from allergic patients were collected before and after subcutaneous immunotherapy and injected into the skin of non-allergic subjects. Skin tests performed in these pre-treated areas showed that sera obtained after AIT were blocking skin reactivity in an allergen-specific manner. Based on these observations, Cooke et al. concluded that there is “the development under treatment of a peculiar blocking or inhibiting type of immune antibody that prevented the action of allergen on the sensitizing antibody,” hence coining the concept of blocking antibodies as a “transferable protective substance” that accounts for clinical improvement after AIT [32]. Loveless characterized the blocking antibodies as IgG antibodies [33]. Blocking antibodies are immunoglobulins of any isotype other than IgE, mostly IgG_1_ and IgG_4_ [34, 35], to some extent also IgG_2_ and IgA [36], that compete with IgE for the binding to the same allergen molecule (Fig. 1) and therefore prevent allergen-induced activation of mast cells and basophils [37], as well as IgE-facilitated allergen presentation [38, 39]. In a pivotal study, Loveless could demonstrate the correlation between the degree of clinical improvement after AIT and the blocking capacity of sera obtained from patients after vaccination by calculating the amount of allergen that can be neutralized per ml of such sera in skin studies [40].

In an attempt to develop laboratory tests that mimic the reduction of in vivo sensitivity to allergens, the histamine-release test was used to demonstrate the development of blocking antibodies in the course of AIT by their ability “to react with antigen in the fluid phase, thereby diminishing the anaphylactic release of histamine from leukocytes,” in addition to tests showing the competition of blocking antibodies with allergen-specific IgE for binding to allergen [41]. These early findings suggest that the measurement of blocking antibodies which interfere with the IgE recognition of allergens can be used as universal biomarker to assess the clinical efficacy of AIT.

## Limitations of the assessment of blocking antibodies as biomarker for clinical efficacy of AIT

There are several reasons why the assessment of blocking antibodies cannot totally mimic clinical efficacy of AIT. First of all, allergic patients suffer not only from immediate allergic reactions caused by allergen/IgE-induced mast cell and basophil activation. In fact, it has been shown that non-IgE-reactive allergen peptides can also cause allergic inflammation by activation of allergen-specific T cells [42]. Furthermore, AIT using allergen extracts which are generally derived from natural sources are often of poor quality and do not induce protective antibody responses against each of the allergens recognized by the individual patient. Additionally, one must bear in mind that only a portion of IgG antibodies induced by allergen extracts is directed against allergens and has the ability to block IgE binding [39]. Moreover, pivotal endpoints used as markers for clinical efficacy of AIT such as symptom and medication scores are influenced by allergen exposure and thus may vary with the extent of natural allergen load, epithelial barrier function and individual habits of the patient and therefore may not reflect efficacy of AIT. Furthermore, other aspects like prediction of clinical efficacy of AIT and sustainability of its effects, as well as availability of biomarkers providing a rationale on how to proceed in cases of no or poor improvement of the patient’s clinical condition after AIT are still unmet needs [43, 44].

It is therefore not surprising that there is an ongoing search for better biomarkers but the results to date are not very promising. Some authors have investigated the association of the clinical outcome of AIT with changes in serum levels of particular cytokines, especially interleukin (IL)-10. The transient increase in serum levels of IL-10 during the early phases of AIT was confirmed by several authors [45, 46], but no clear and significant association with clinical efficacy could be demonstrated so far [47]. This is not unexpected since changes in cytokine levels do not reflect tolerance induction to all of the components the patient has demonstrated allergic sensitivity and thus, IL-10 is not a suitable marker for clinical efficacy of AIT. Attempts to correlate clinical parameters with therapy-induced changes in allergen-specific antibodies of certain classes or subclasses (IgE, IgG, IgG_1_, IgG_4_, or IgA) yielded conflicting results [48–52]. Ratios of allergen-specific IgE to total IgE [53] or of allergen-specific IgG_4_ to IgG_1_ [54] showed better results in terms of prediction of or correlation with clinical outcome in some studies which, however, could not be confirmed in subsequent trials. Likewise, efforts to adopt techniques for assessing reactivity of allergen-specific T cells for the monitoring of AIT did not prove suitable for routine in vitro tests and do not reflect immediate-type symptoms caused by mast cell and basophil activation [55, 56].

## In vivo surrogate markers for clinical efficacy of AIT

There are several in vivo tests that were suggested for monitoring of AIT, e.g., nasal, bronchial, or conjunctival provocation and controlled allergen exposure in challenge chambers [57]. In vivo tests offer the possibility to investigate the improvement of patients in the course of AIT in a longitudinal manner by comparing sensitivity before and after treatment, and it has been shown that meaningful differences can also be demonstrated between actively and placebo-treated patients even in small groups of subjects. However, these in vivo tests are laborious, require proper equipment and experience. They are suited mainly for clinical trials rather than the assessment of individual patients in clinical routine. There is therefore a demand for simple laboratory tests which can be used to assess the effects of AIT on allergen-specific immune responses. Such tests are needed because they help to understand why certain patients do not respond to treatment, for example, by revealing lack of induction of an IgG response to allergens against which the patient is sensitized. This is an important piece of information since AIT is a time-consuming treatment conducted over several years to ensure sustainable effects, and it may also cause side effects. Lack of induction of a protective immune response by a certain allergen extract can thus be detected which allows to discontinue or adjust the treatment.

## Towards blocking antibodies as biomarker for the effect of AIT

There are several possible ways to measure the blocking effect of AIT-induced antibodies as a marker for the AIT effects on IgE-mediated mast cell and basophil activation, as well as on IgE-mediated T cell activation. In fact, different types of cell-based in vitro assays were developed that mimic the effects of blocking IgG on IgE-mediated mechanisms occurring in vivo, like basophil activation tests performed in the presence of IgG [58–61], inhibition of allergen-induced T cell proliferation and cytokine release using patients’ cells [38] or the facilitated allergen binding (FAB) assay that uses a CD23-expressing B cell line together with an IgE-containing indicator serum [62, 63]. Using these tests, the effects of blocking antibodies on immediate-type effector cell responses that are based on mast cell and basophil activation, as well as their impact on late phase responses by T cell activation can be studied.

Schmid et al. were successful in demonstrating that a favorable clinical outcome can be predicted by early reduction in basophil sensitivity during AIT to grass pollen [64], and James et al. in demonstrating that the persistence of the blocking effect of allergen-specific IgG antibodies in the FAB test correlates with sustained clinical benefit from AIT [34]. However, in contrast to serological tests, these assays are laborious and difficult to standardize [63, 65] and therefore, implementation for routine monitoring of AIT may be difficult and requires defined allergen preparations.

**Fig. 2:**
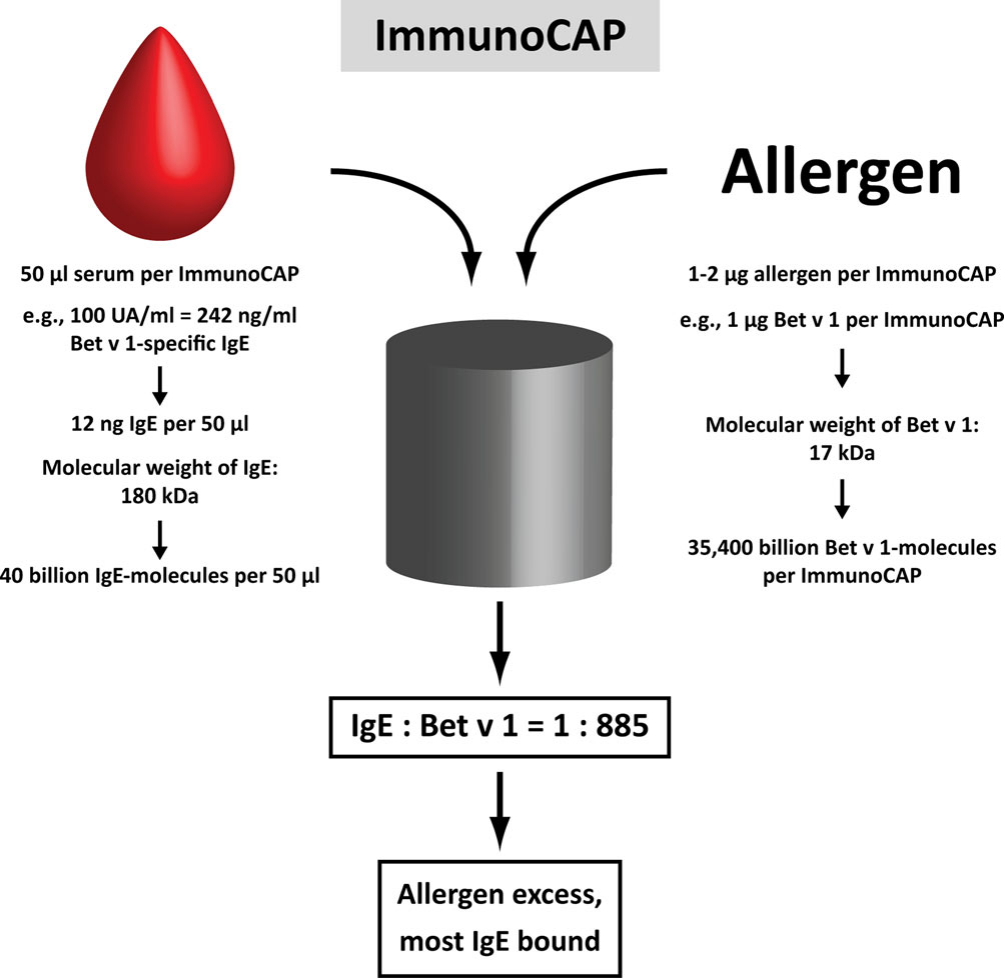
Example of the calculation of the ratio of allergen-specific IgE and allergen molecules in a test system using allergen excess (e.g., ImmunoCAP).

**Fig. 3:**
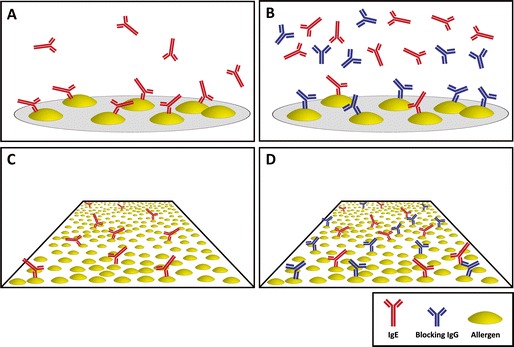
Samples that are devoid of blocking IgG antibodies (*blue*) yield similar results for detection of IgE (*red*) in serological testing by microarray (**a**) and by tests that employ a large amount of allergen (*yellow*) per assay, like ImmunoCAP (**c**). By contrast, in presence of blocking IgG, competition between IgE and blocking IgG antibodies for binding to the same allergen molecule reduces IgE levels detected by microarray containing low amounts of allergen (**b**) but not in tests using large quantities of allergen (**d**).

In this context, it must be emphasized again that, since any allergen extract used for AIT may induce IgG to allergens as well as to non-allergenic molecules contained in the vaccine, quantification of IgG response to the complete vaccine is of minor significance for the assessment of a therapeutically relevant immune response to AIT treatment [39]. The development of tests using purified recombinant or natural allergen molecules allowed for specific quantification of the fraction of IgG directed against the allergen proper, which improved accuracy of monitoring. However, it was not possible to differentiate blocking from non-blocking IgG antibodies [48, 52]. The reason for that is that in most tests used for the quantification of allergen-specific antibodies, like ImmunoCAP (Thermo Fisher Scientific, Uppsala, Sweden) or ELISA, an excess of allergen molecules is employed when compared with levels of antibodies in patients’ sera which bind to that particular allergen (Fig. 2). The ImmunoCAP test, for instance, comprises 1–2 μg of allergen. In the case of Bet v 1 from birch pollen, for example, 1 μg corresponds to approximately 35,400 billion molecules (Fig. 2, right). If a serum sample with, e.g., 100 UA/ml (units allergen-specific IgE per milliliter) of Bet v 1-specific IgE (equals approximately 242 ng/ml [66]) is tested, 50 μl of serum that are applied to the test system contain ca. 40 billion molecules of Bet v 1-specific IgE (Fig. 2, left). Hence, the allergen-to-IgE ratio in the test system amounts to ca. 900:1 or, in other words, every single IgE molecule in that serum sample has almost 900 binding sites to “choose from” for binding to the ImmunoCAP. This surplus of binding sites allows almost quantitative IgE binding (Fig. 3c), even in presence of high titers of blocking IgG antibodies (Fig. 3d) [67•]. Therefore, these tests are well suited for the quantification of humoral immune responses to allergen molecules, both of the IgE and IgG isotype.

However, amounts of allergen that are incorporated by patients following allergen contact are extremely low [68–70] and, accordingly, competition between IgE and IgG for the limited number of binding sites takes place. This is why tests employing an excess of allergen molecules for antibody measurement do not reflect in vivo conditions which is an important reason for the poor correlation of test results with clinical outcome that is observed in many patients.

In an attempt to measure the blocking effect of AIT-induced non-IgE antibodies, Würtzen et al. [71] adopted a test protocol for IgE detection (Advia Centaur, Siemens Healthcare GmbH, Erlangen, Germany). In this single-plexed liquid phase assay, patient’s IgE is captured by paramagnetic beads that are covered with anti-IgE antibodies as a first step. Allergen-specific IgE is then quantified by addition of a molar excess of labeled allergen [72]. By omitting the washing step that removes serum IgG prior to additon of allergen, the authors could measure the blocking effect of therapy-induced IgG on IgE binding to allergens. Results obtained with the modified protocol showed good correlation with changes in histamine release and FAB assays, indicating that serological tests can yield results that are equivalent to those from cell-based systems. However, no significant correlation with clinical parameters like symptom or medication scores could be demonstrated, presumably due to the limiting factors mentioned above that apply for assays measuring blocking antibodies as surrogate for clinical efficacy of AIT [71].

## Allergen microarrays can detect the induction of blocking antibodies

In 2002, Hiller et al. published the first study illustrating the possibility of employing protein microarray technology for allergy diagnosis [73]. At that time, most of the prevalent allergen molecules were available as well defined recombinant or purified natural proteins and so the first allergen microarray, Immuno Solid-phase Allergen Chip (ISAC, VBC Genomics, Vienna, Austria, today Thermo Fisher, Uppsala, Sweden), was “born” and soon approved for allergy diagnosis. This multiplexed test allows the detection of IgE or IgG antibodies to a large number of allergen components in one step, consuming only minute amounts of serum. Since the release of the first version of ISAC, substantial improvements were made regarding the allergens included on the array, coupling chemistry and detection systems so that the sensitivity of the test could be further increased. The clinical value of multiplexed serological tests for component resolved diagnosis (CRD) in terms of improved accuracy of diagnosis and prescription of AIT has been reviewed [22, 74].

**Fig. 4:**
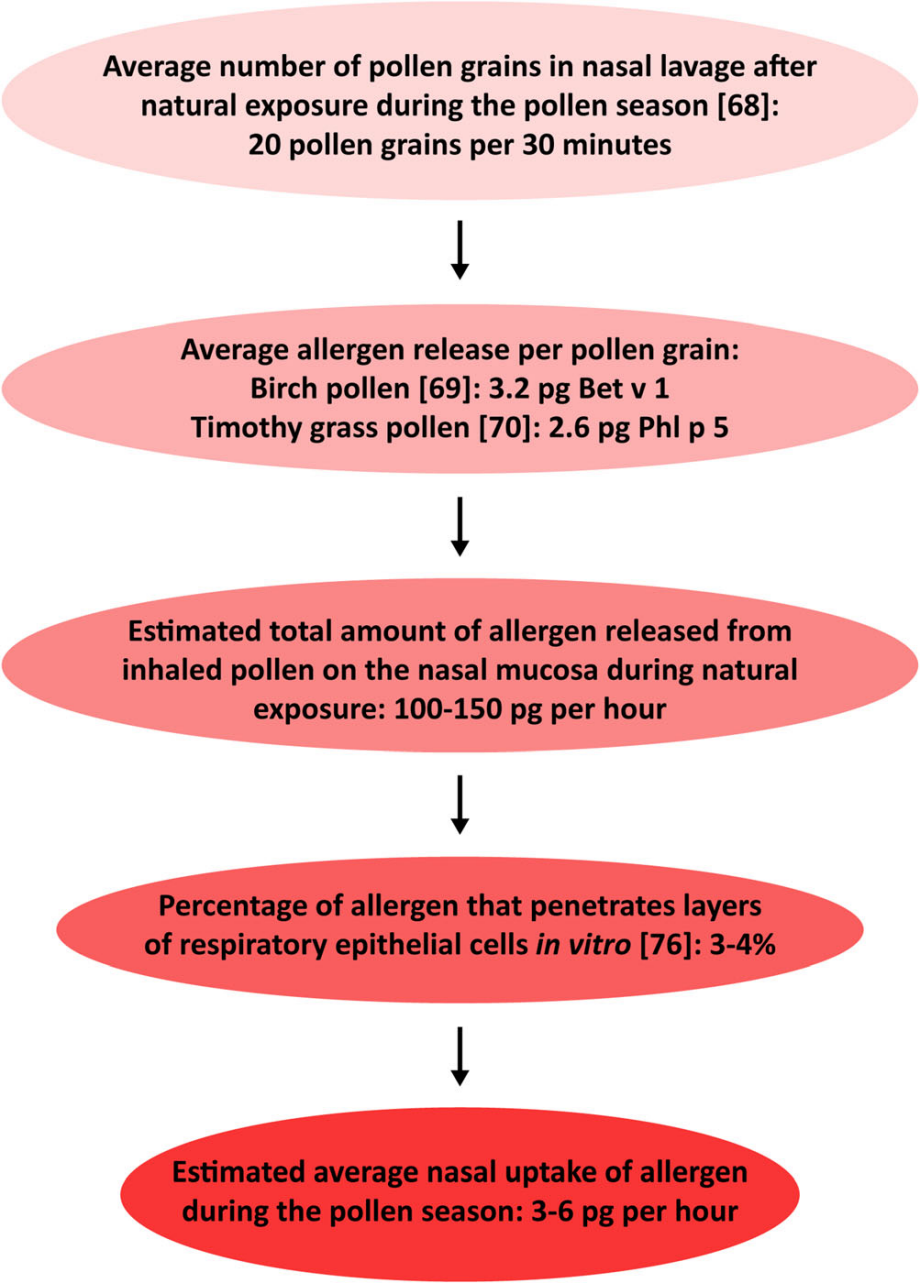
Approximation of allergen uptake by the nasal mucosa during “natural” seasonal pollen exposure.

One major difference between microarrays and most other serological test systems is that on an allergen chip, the amount of protein immobilized per spot lies in the range of 100 fg (i.e., approximately 1 attomol or 600,000 molecules per spot). This is approximately 10,000,000 times less when compared with, e.g., the ImmunoCAP system, where 1–2 μg of allergen is used per assay. Due to this markedly reduced number of allergen molecules per test, microarrays mimic in vivo conditions more closely where also only few allergen molecules enter via the skin or the mucosa and combine with IgE and IgG antibodies. On average, around 20 pollen grains are found in the nasal mucosa after 30 min of “natural” exposure during the pollen season [68] (Fig. 4). For example, a birch pollen grain contains approximately 6 pg of Bet v 1 [75]. Allergen release from pollen into ambient air allowed to calculate an average discharge of around 3.2 pg of Bet v 1 per birch pollen grain [69]. Similar results were found for timothy grass pollen with 2.6 pg of Phl p 5 released per pollen grain [70]. Coupled to small airborne particles derived from the pollen themselves, allergen molecules can also reach lower airways [75]. Considering the fact that only a few percent of allergen molecules will permeate the mucosa [76], these findings indicate that ratios between levels of allergen molecules and allergen-specific IgE and IgG that occur at sites of allergen incorporation through mucosal surfaces after discharge of allergens by inhaled pollen grains or following allergen-delivery by small inhaled particles, seem to be astoundingly well reflected by serological testing using allergen chips. The same is true for skin prick testing where an average volume of 16 nanoliters enters the skin [77] which leads to incorporation of around 10–300 pg of allergen, depending on allergen-concentration in the test solution [11, 12].

Hence, like in vivo, where blocking antibodies may lead to inhibition of IgE binding to allergen molecules which causes reduction of mast cell and basophil activation in tissues [32, 33, 35], IgE-signal intensities measured by allergen chips may be reduced by blocking antibodies as well (Fig. 3a, b). This was recently demonstrated in a study using a customized version of ISAC, the MeDALL chip [67•]. In these experiments, a monoclonal human IgE antibody specific to Bet v 1, the major birch pollen allergen, was mixed with increasing concentrations of a monoclonal IgG antibody sharing identical variable domains, i.e., binding to exactly the same epitope. These experiments clearly showed that the allergen chip can detect the blocking effect of allergen-specific IgG with high sensitivity, even in cases of low levels of specific IgE, which is in contrast to the ImmunoCAP system where no changes were observed up to a 100-fold excess of blocking IgG. Using the MeDALL chip, it was also proven that the sensitivity of microarrays for IgE detection is equal or even superior to technologies using allergen excess [67•, 78].

Until now, other platforms for multiplexed serological allergy diagnosis have been developed, e.g., FACS-based microbead arrays [79], Microtest (London, UK), MARIA (Indoor Biotechnologies, Virginia, USA), and others, some of which already were approved for clinical use. However, the capacity of these tests to measure the blocking effect of AIT-induced non-IgE antibodies remains to be investigated.

## Monitoring of AIT by allergen microarray

Based on the strong evidence for the role of blocking IgG in AIT and on the aforementioned findings on the detection of the blocking effect of IgG by microarray, results from clinical trials were published recently that corroborate the potential usefulness of allergen microarrays for the monitoring of AIT.

Wollmann et al. reported results from IgE and IgG measurements by ImmunoCAP and ISAC in sera from patients who were vaccinated with two hypoallergenic Bet v 1-derivatives [80••]. Both serological test systems were concordant in detecting the induction of allergen-specific IgG in treated subjects, but differed in results from IgE measurements. While in sera obtained from treated individuals, a boost of Bet v 1-specific IgE was observed by ImmunoCAP, analysis of the same samples by ISAC revealed a decrease of Bet v 1-specific IgE, indicative for the blocking activity of AIT-induced IgG. In this AIT trial, nasal provocation tests were performed as a surrogate for clinical sensitivity. For the population studied, a drop in specific IgE had high predictive value for an increase in nasal tolerance in provocation testing.

In another clinical study, Schmid et al. analyzed sera from 24 grass-pollen-allergic patients suffering from allergic rhinoconjunctivitis, 18 of whom were subjected to subcutaneous immunotherapy (SCIT) with grass pollen extract and 6 receiving symptomatic medication only [81••]. In accordance with previously published data, an increase in IgG_4_ and a decrease in IgE specific to grass pollen allergens were demonstrated by ISAC measurements whereas in ImmunoCAP both IgE and IgG_4_ rose. The authors observed that in the treated subjects baseline levels and reactivity patterns of grass pollen allergen-specific IgE allowed the prediction of the specificities and the magnitudes of IgG_4_ responses to the respective grass pollen components. In addition, strong correlations between IgE and IgG_4_ levels that were measured by ISAC with findings from basophil activation tests, FAB assays and with symptom scores were demonstrated [81••].

In an approach to further dissect the immune response to allergen molecules, microarrays comprising overlapping peptides that cover the complete amino acid sequence of different allergen molecules were developed. This technology was used by Vickery et al. for monitoring of an oral immunotherapy (OIT) trial aiming at the induction of tolerance to peanut allergens [82•]. During 4 years of OIT, patients mounted an increasing spectrum of IgG_4_ specificities with growing signal intensities that was paralleled by a decrease of IgE levels but not by changes of IgE-reactivity patterns. Furthermore, the authors observed an induction of IgG to particular peptides that were described in a previous study to distinguish peanut-sensitized subjects with symptoms from sensitized individuals without clinical symptoms upon peanut ingestion [83], indicating that it may be possible to precisely predict the success of AIT by peptide microarrays. The same principle was applied by Savilahti et al. in patients suffering from cow’s milk allergy to predict the natural course of the disease [84], to forecast the outcome of OIT to cow’s milk allergy and to differentiate serologically between successfully treated patients and non-responders [85••]. These results could be replicated by Martínez-Botas et al. who, in addition, could identify IgE-reactivity patterns to peptides from cow’s milk allergens that allowed to distinguish high- from low-risk patients in terms of frequencies of allergic reactions during treatment, consumption of rescue medication, and time needed to achieve clinical improvement [86].

These studies provide evidence for the feasibility of microarray-based serological findings to be used as surrogate marker for the monitoring and the prediction of clinical efficacy of AIT. Even though data are still restricted to few allergen sources, it was already demonstrated that the concept can be applied to both respiratory and food allergies.

For the serological monitoring of AIT, availability of baseline values is essential to evaluate therapeutically induced changes in antibody-reactivity patterns and levels. Therefore, a serum sample should be collected from every patient before start of AIT and stored in a freezer. This would enable the clinician to evaluate the development and alteration of the immune response to the allergens at a later point. This could be particularly useful in patients for which immunotherapy has not achieved the desired clinical effects, i.e., lack of clinical improvement or even worsening of symptoms. For example, detection of a decrease of IgE signals, albeit still too weak, should endorse the physician’s decision to pursue AIT, aiming at a further reduction of IgE levels detected. By contrast, no change in IgE levels at all or absence of IgG induction to those allergen molecules the patient is sensitized to could indicate that the patient’s immune system does not respond to the vaccine or a lack of particular allergen components in the extract used for treatment [12]. These findings could help the clinician to decide if AIT should be continued, modified, e.g., by using a vaccine from a different producer, or even discontinued in the individual patient.

Allergen microarrays are continuously subjected to technical modifications that shall further enhance their clinical versatility. First, by optimization of the panel of allergen components that are represented on the chip, accuracy of prescription of AIT will be even improved. Knowing the patient’s molecular sensitization pattern may allow to estimate the propensity of AIT to be beneficial, especially in case of vaccination with extracts that are frequently devoid of particular, clinically relevant allergens as those from house dust mite [14]. Second, by employing peptides that replace or supplement complete allergen molecules on the microarray, immune responses to those epitopes that are involved in the initiation of symptoms could be distinguished from clinically less relevant IgE reactivities. Third, advances in chip production and in protocols for serum analysis should allow reduction of average costs per analysis.

In summary, if microarray-based algorithms for the prediction and monitoring of the course of AIT can be established and replicated for additional allergen sources, it will be possible to distinguish patients who are likely to benefit from AIT from those who most probably will not. If this can be achieved early in the course of AIT or even before start of vaccination, this patient-tailored approach is a classical example of precision medicine that would help to improve prescription and monitoring of AIT and thus reduce the burden of unnecessary risks of adverse effects and costs and to safe time, hence creating benefits for patients, doctors, and the health care system.
